# Risk Factors for Rivaroxaban-Related Bleeding Events—Possible Role of Pharmacogenetics: Case Series

**DOI:** 10.3390/pharmacy11010029

**Published:** 2023-02-05

**Authors:** Livija Šimičević, Ana Marija Slišković, Majda Vrkić Kirhmajer, Lana Ganoci, Hrvoje Holik, Jozefina Palić, Jure Samardžić, Tamara Božina

**Affiliations:** 1 Division of Pharmacogenomics and Therapy Individualization, Department of Laboratory Diagnostics, University Hospital Centre Zagreb, 10000 Zagreb, Croatia; 2 Department of Cardiovascular Diseases, University Hospital Centre Zagreb, 10000 Zagreb, Croatia; 3 Department of Internal Medicine, School of Medicine, University of Zagreb, 10000 Zagreb, Croatia; 4 Department of Internal Medicine, General Hospital Dr Josip Benčević, 35000 Slavonski Brod, Croatia; 5 Department of Medical Chemistry, Biochemistry and Clinical Chemistry, School of Medicine, University of Zagreb, 10000 Zagreb, Croatia

**Keywords:** drug safety, interactions, multidisciplinary, pharmacogenetic, risk factors, rivaroxaban, rivaroxaban-related bleeding

## Abstract

Non-vitamin K antagonist oral anticoagulants’ interindividual trough concentration variability affects efficacy and safety, especially in bleeding events. Rivaroxaban is metabolised via CYP3A4/5-, CYP2J2-, and CYP-independent mechanisms and is a substrate of two transporter proteins: ABCB1 (MDR1, P-glycoprotein) and ABCG2 (BCRP; breast-cancer-resistance protein). The polymorphisms of these genes may possibly affect the pharmacokinetics of rivaroxaban and, consequently, its safety profile. Rivaroxaban variability may be associated with age, liver and kidney function, concomitant illness and therapy, and pharmacogenetic predisposition. This case series is the first, to our knowledge, that presents multiple risk factors for rivaroxaban-related bleeding (RRB) including age, renal function, concomitant diseases, concomitant treatment, and pharmacogenetic data. It presents patients with RRB, along with their complete clinical and pharmacogenetic data, as well as an evaluation of possible risk factors for RRB. Thirteen patients were carriers of *ABCB1*, *ABCG2*, *CYP2J2*, and/or *CYP3A4*/5 gene polymorphisms. Possible drug–drug interactions with increased bleeding risk were identified in nine patients. Six patients had eGFR <60 mL/min/1.73 m^2^. Our data suggest a possible role of multiple factors and their interactions in predicting RRB; however, they also indicate the need for further comprehensive multidisciplinary research to enable safer use of this product based on a personalised approach.

## 1. Introduction

The global anticoagulant market has undergone continuous growth that is mostly driven by an ageing population as well as an increasing cardiovascular, diabetic, and cancer population. Warfarin, a vitamin K antagonist, is the most commonly used oral anticoagulant. However, non-vitamin K antagonist oral anticoagulants (NOACs) have progressively become more prescribed antithrombotic drugs. During the COVID-19 pandemic, ongoing clinical trials with anticoagulant treatment are also influencing this market. Consequently, the NOACs market is expected to grow more progressively in the upcoming years [1]. NOACs’ interindividual trough concentration (C_trough_) variability affects efficacy and safety, especially in bleeding events. This variability may be associated with age, liver and kidney function, concomitant illness and therapy, and pharmacogenetic predisposition [2,3]. The present increase in the prescription of anticoagulants will probably correlate with an increase in unexpected bleeding complications. This is already noticeable in the number of NOACs that are implicated in almost 40% of cases of emergency room visits due to oral anticoagulant-associated bleeding [4].

Research findings to date suggest that drug interactions, as well as pharmacogenetics, are major contributors to the variability in NOAC plasma concentrations; however, the evidence is insufficient to translate these findings into clinical practice [5]. Therefore, it is necessary to develop a risk score that would better reflect the real-life situation of patients with comorbidities and polytherapy in order to predict interactions of multiple drugs and genes resulting in more frequent adverse drug reactions (ADRs) and hospitalizations, thus increasing expenditures to the healthcare system.

Rivaroxaban (RIVA) is a selective, reversible, factor Xa inhibitor indicated in the treatment of atrial fibrillation (AF), coronary artery disease (CAD), indefinite anticoagulation peripheral artery disease (PAD), thromboprophylaxis, andvenous thromboembolism (VTE) and in reducing the risk of recurrent VTE, VTE prophylaxis in acutely ill medical patients, and VTE prophylaxis in total hip or knee arthroplasty [6]. Interindividual C_trough_ variability of RIVA could be related to age, kidney function, concomitant therapy, and also pharmacogenetic predisposition. RIVA is subject to additional drug monitoring, thus enabling rapid recognition of new safety information [7,8]. It is metabolised via CYP3A4/5, CYP2J2, and CYP-independent mechanisms and is a substrate of two transporter proteins: ABCB1 (MDR1, P-gp; P-glycoprotein) and ABCG2 (BCRP; breast cancer resistance protein) [7]. Due to its pharmacokinetics, strong dual CYP3A4 and P-gp inhibitors can increase RIVA bioavailability, while strong CYP3A4 inducers and/or P-gp inducers can decrease it [9]. The polymorphisms of these genes may possibly affect the pharmacokinetics of RIVA and, consequently, its safety profile.

This case series includes patients who have experienced RIVA-related bleeding along with their complete clinical and pharmacogenetic data, as well as an evaluation of possible risk factors for RIVA-associated bleeding. 

## 2. Case Series Presentation

The presented cases are subjects of a more extensive prospective nested case–control study “Pharmacogenomics in Prediction of Cardiovascular Drugs Adverse Reaction—PGx CardioDrug” (Appendix A). These sixteen patients (nine females, seven males; median age 73 years, range 61–80) have been consequently included in the study as RIVA-related bleeding cases: gastrointestinal (GI) (N = 9), epistaxis (N = 5), haematuria (N = 1), and gynaecological (N = 1). After cessation of RIVA, all bleeding ceased. The average RIVA daily dose was 17.5 mg (range 5–20 mg) and was administrated according to the following indications: AF (N = 13), deep vein thrombosis (DVT) (N = 2), and PAD (N = 1). Bleeding occurred between 1 and 12 months following the introduction of RIVA. The subjects’ demographic, clinical, and genotype data with possible known drug–drug interactions (DDIs) are summarised in Table 1.

In total, these subjects had, on average, four concomitant diseases (range 1–8 diseases) and eight concomitant drugs (range 2–17 drugs). For the investigation of DDIs, the Lexicomp^®^ Clinical Decision Support System [10] was applied. Possible known DDIs such as as possible bleeding risk factor were identified for seven concomitant drugs in nine patients and in a total of 11 drug–drug interactions (4/11 B, No action needed; 1/11 C, Monitor therapy; 6/11 D, Consider therapy modification) (Table 1). Furthermore, as a second DDI source, we used the DrugBank’s drug–drug interaction checker [11]. According to this database, we identified 34 concomitant drugs with possible interactions with RIVA; 1 major DDI, 15 moderate, and 18 minor DDIs; all of these, along with their possible mechanisms, are presented in Table 2.

The concomitant use of RIVA with certain antiplatelet agents (such as aspirin and clopidogrel) and nonsteroidal anti-inflammatory drugs (such as ketoprofen and indomethacin) is known to increase the risk of bleeding due to a synergistic effect, i.e., an increased anticoagulant effect of RIVA. The concomitant use of RIVA with antidepressants (in this case duloxetine, a combined serotonin and norepinephrine reuptake inhibitor) is known to increase the risk of bleeding due to pharmacodynamic DDIs, thus increasing the anticoagulant effect of RIVA and bleeding risk [10]. The severity of the above-mentioned interactions is described as moderate in the DrugBank [11], but in Lexicomp, the RIVA interaction with aspirin, clopidogrel, ketoprofen, and indomethacin is classified as “D” (Consider therapy modification) [10]. According to NOAC’s Drug–Drug interaction guidance [12], such combinations are recommended only when benefits exceed treatment risk, and even in this case, patients receiving such combined therapy should be reassessed and observed for bleeding. Amiodarone is a CYP3A4 inhibitor that at the same time also inhibits CYP2J2 and P-glycoprotein; thus, it increases RIVA plasma concentrations, and this interaction is classified as major [11]. However, this DDI is classified as “B” (No action needed) in Lexicomp [10].

Renal function was assessed based on the estimated glomerular filtration rate (eGFR) with the CKD-EPI equation [13]. Two patients had stage 1 chronic kidney disease (CKD), i.e., normal renal function (eGFR >90 mL/min/1.73 m^2^), eight patients had stage 2 CKD (slightly reduced eGFR 60–89 mL/min/1.73m^2^), and six had impaired kidney function (eGFR < 60 mL/min/1.73 m^2^). Among these six, two patients had stage 3a CKD (eGFR 45–59 mL/min/1.73 m^2^) and four had stage 3b CKD (eGFR 30–44 mL/min/1.73 m^2^) [14]. Two patients with impaired kidney function were administrated RIVA in a dose of 20 mg/day. 

All presented subjects were genotyped for relevant ADME gene variants: CYP2J2*7 (rs890293) and rs11572325, CYP3A4*1B (rs2740574), CYP3A4*22 (rs35599367), CYP3A5*3 (rs776746), *ABCB1* (*c.*1236C>T [rs1128503], *c*.2677G>T/A [rs2032582], *c*.3435C>T [rs1045642], *c.*2482-2236G>A [rs4148738], *ABCG2 c*.421C>A (rs2231142). Pharmacogenetic analyses were performed by specific TaqMan^®^ DME and SNP Assays on a 7500 Real-Time PCR System (Applied Biosystems, Thermo Fisher Scientific, Waltham, MA, USA). Demographic, medical, and laboratory data (Table 1) show that 13 subjects had at least one variant allele that may possibly influence RIVA pharmacokinetics in terms of higher bleeding risk. Four subjects were CYP2J2*7 heterozygous carriers, six were *CYP2J2* rs11572325 heterozygous carriers, two patients were CYP3A4*22 carriers (*1/*22 and *22/*22), three were CYP3A5*3 heterozygous (expressers), two patients had the *ABCB1* 1236T-2677T-3435T-rs4148738A homozygous variants haplotype, three patients had *ABCG2* 421CA (heterozygous) and one *AA* genotype (homozygous variant carrier). Only three patients had all wild-type investigated pharmacogene variants. 

We grouped our subjects according to the number of possible bleeding risk factors into five groups. 

Group 1, with pharmacogene ABCB1 rs4148738 GG genotype that may influence RIVA pharmacokinetics in terms of higher bleeding risks (one risk factor): three patients, two males (64 and 68 years old; they both experienced GI bleeding), and one female (66 years old; with epistaxis) (Table 3). These patients experienced bleeding 6, 9, and 12 months following the introduction of RIVA. 

Group 2) with several known pharmacogene variants that may influence RIVA pharmacokinetics in terms of higher bleeding risks (one risk factor): three patients, two females (69 and 72 years old; both with epistaxis) and one male (66 years old; with GI bleeding and anaemia). All three patients experienced ADR 4-5 months following the introduction of RIVA. They were carriers of CYP2J2*7, CYP3A4*22, *ABCG2* c.421A and *ABCB1* 1236T-2677T-3435T-rs4148738G variants with a possible association with increased RIVA concentration (Table 4).

Group 3, with possible DDI as a possible bleeding risk factor and with known pharmacogenes variants that may influence RIVA pharmacokinetics in terms of higher bleeding risks (two risk factors). In this group, we have four patients: two males (66 and 78 years old, with haematuria and epistaxis, respectively) and two females (61 and 75 years old, with gynaecological bleeding and melena, respectively) (Table 5). Patient N1 shown in Table 5 had RIVA–propafenone interaction. Propafenone is a known P-glycoprotein (ABCB1) inhibitor and CYP3A4 substrate, and its mechanism of interaction is based on ABCB1 inhibition. According to DrugBank (Table 2), the patient had five possible minor RIVA DDIs (diazepam, finasteride, folate, metoprolol, and tamsulosin), based on increased RIVA concentration, decreased renal excretion, or competition for metabolism plus one moderate RIVA DDI with propafenone. Moreover, this patient was a carrier of all four variant alleles of *ABCB1* with a possible influence on peak levels of rivaroxaban with increased RIVA concentration. Patient N2 in shown Table 5 experienced extreme gynaecological bleeding one month after the introduction of RIVA 10 mg/day. She also had in-therapy ASA (DDI with increased bleeding risk), plus bisoprolol and pantoprazole (both CYP3A4 substrates and ABCB1 inhibitors; Table 2). This patient was a homozygous carrier of CYP3A4*22 (a poor metaboliser), and a heterozygous carrier of all variants of CYP2J2*7 and *ABCB1*. This pharmacogenetics finding may possibly cause increased RIVA concentration. Patient N3 had as concomitant therapy clopidogrel (DDI with increased bleeding risk), plus bisoprolol and pantoprazole (both CYP3A4 substrates and ABCB1 inhibitors) and amiodarone (CYP3A4 and ABCB1 substrate and inhibitor) (Table 2). He was identified as a heterozygous carrier of all four variant alleles of *ABCB1*. Patient N4 experienced epistaxis 6 months following the introduction of RIVA at only 5 mg/day. His concomitant therapy included ASA, but he is furthermore a heterozygous carrier of CYP2J2*7 and a homozygous carrier of all variants of *ABCB1,* and that, together with DDI, may possibly cause increased RIVA concentration.

Group 4, with decreased kidney function and with known pharmacogenes variants that may influence RIVA pharmacokinetics in terms of higher bleeding risk (two risk factors): one patient (presented in Table 6) was female, 76 years old. She has stage 3b CKD with eGFR 34 mL/min/1.73 m^2^. Additionally, she is a heterozygous carrier of *ABCG2 c*.421A with a decreased function that may possibly cause increased RIVA concentration.

Group 5, with decreased kidney function, possible DDI, and known pharmacogene variants that may influence RIVA pharmacokinetics in terms of higher bleeding risk (three risk factors). In this group, we have five patients (Table 7): three females (74, 75 and 80 years old) and two males (66 and 75 years old). Patient N1, with stage 3b CKD, experienced GI bleeding 3 months following the introduction of RIVA at 15 mg/day. Her concomitant therapy included, among other drugs, clopidogrel, duloxetine, pantoprazole, and bisoprolol, with possible increased RIVA concentration DDIs. Furthermore, we identified homozygosity for all variants of *ABCB1* and heterozygosity for *ABCG2 c.*421A. Patient N2 also had stage 3b CKD and experienced GI bleeding and anaemia one year following the introduction of RIVA. He has heterozyogosity for all variants of *ABCB1* and, as concomitant treatment, indomethacin, bisoprolol, and pantoprazole as possible drugs interacting with RIVA. Patient N3 had also stage 3b CKD. She experienced epistaxis three months following the introduction of RIVA. Her medical documentation revealed that she was taking 17 drugs plus RIVA. Additionally, she is heterozygous for CYP2J2*7 and a heterozygous carrier of all variants of *ABCB1*. Patient 4 had stage 3a CKD and had only amiodarone, pantoprazole, and perindopril in concomitant therapy. All these drugs can interact with RIVA and increase RIVA concentration. This patient’s pharmacogenetics result is that hee is a heterozygous carrier of all variants of *ABCB1*. Patient N5 also had stage 3a CKD. Her concomitant therapy includes amiodarone, atorvastatin, and metformin, with potential interaction with RIVA. She is a heterozygous carrier of *ABCB1 c*.1236T and *c*.3435T.

This real-world data from 16 patients with adverse bleeding events indicates that the pharmacogene variant alleles of enzymes and transporters of the metabolic pathway of RIVA are associated with either elderly age, DDIs, and decreased renal function, separately or in combination, in occurrence of RIVA-related bleeding. 

## 3. Discussion

This case series is the first, to our knowledge, that presents multiple risk factors for RIVA-related bleeding, including age, renal function, concomitant diseases, and concomitant treatment and pharmacogenetics data. Pharmacogenetic data represent the cornerstone of personalised medicine [15]. However, data from the conducted pharmacogenetics studies on NOACs did not draw a clear conclusion; rather, they only scratched the surface of the issue of NOACs pharmacokinetics [8].

CYP2J7 enzyme is, together with CYP3A4, the key rivaroxaban (RIVA) hydroxylation path and metabolism enzyme. Zhao et al. have recently published a systematic evaluation of CYP isoforms’ participation in the metabolism of RIVA and demonstrated for the first time the main role of CYP2J2 in RIVA metabolism (41.1%). The contribution of CYP3A4 was much lower in that study (27.3%) [16]. This finding is very important for possible DDIs and increased bleeding risk. The most recognized functional *CYP2J2* variant is CYP2J2*7, with a general frequency of 2.1%-17% [17,18]. In Caucasians, the *7 (rs890293) is associated with 40% lower enzyme expression, but without considerable change in CYP2J2 activity [19]. Nakagawa et al. investigated the effect of rs890293 on trough concentrations of rivaroxaban, but in the study, there was no CYP2J2*7 homozygous carriers and this question remains open [20]. Intron variant rs11572325 does not have an effect on CYP2J2 expression and/or activity, although rs11572325 shows positive linkage disequilibrium (LD) with the rs890293 (*7) associated with reduced levels of CYP2J2 epoxygenase metabolites in vivo [21].

CYP3A4 is involved in the metabolism of common drugs. It is known that CYP3A4*22 carriers have a lower enzyme activity than CYP3A4. The frequency of the intronic *22 allele in Europeans is the highest compared to that of other populations (5%) [22,23]. This polymorphism is significantly associated with reduced CYP3A4 enzyme activity [24,25]. The influence of CYP3A4*22 on pharmacokinetics and pharmacodynamics of NOACs, including RIVA, was investigated due to evidence of the association between CYP3A4 activity and RIVA concentration. The influence of CYP3A4*22 on the pharmacokinetics and pharmacodynamics of NOACs, including rivaroxaban, was investigated due to evidence of the association between CYP3A4 activity and rivaroxaban concentration. Sychev et al. found that the peak and trough RIVA concentrations depended on *CYP3A4* activity [26]. Another *CYP3A4* variant is in a promoter region (CYP3A4*1B, CYP3A4*1.001, rs2740574), and its effect still requires investigation, but it seems it leads to enhanced CYP3A4 activity in combination with the expressor CYP3A5*1 [27,28]. 

For CYP3A5, Sychev et al. aimed to evaluate the effect of the CYP3A5*3 polymorphism on rivaroxaban pharmacokinetics among patients undergoing total hip and knee replacement but no difference was found [29]. A recently published real-world study also investigated the effect of, among others, the CYP3A5*3 variant with bleeding or thromboembolic events of NOACs and did not find an association [5].

P-glycoprotein (P-gp, ABCB1) exerts a protective and excretory function by limiting the intracellular uptake and retention of numerous xenobiotics as well as endobiotics [30]. This transporter has a very important function at the intestinal barrier in the first-pass elimination of a wide range of per os drug substrates. Furthermore, it contributes to the active elimination of molecules from the systemic circulation at the proximal tubules of the kidney [31]. The *ABCB1* genetic variants may have caused the increased concentration of P-gp substrate drugs due to altered active secretion [7,32]. Most research has been performed on three polymorphisms: rs112853 (exon 12 *c*.1236C>T), rs2032582 (exon 21 *c*.2677G>T/A), and rs104566642 (exon 26 *c.*3435C>T) [33]. These variants are in high linkage disequilibrium (in the Caucasian population, approximately 70%) and are recorded as either CGC or TTT haplotypes. Several studies have investigated the effect of ABCB1 polymorphisms on RIVA concentration, bleeding, and thromboembolism events. In 2016, a case of rivaroxaban-induced hemorrhage in a patient with homozygous variant TT genotype for *c.*2677G>T and *c.*3435C>T was published [34]. Another published study did not found an association of these polymorphisms with peak rivaroxaban concentrations [35]. The results of a systematic review and meta-analysis presented elevated RIVA peak concentrations for *c*.2677G>T and *c.*3435C>T homozygous variant carriers [36]. An interesting finding was published of about three cases of major bleeding associated with high RIVA concentration. All three patients were carriers of heterozygous variants of *c*.1236C>T, *c*.2677G>T, and rs4148738 (*c*.2482-22236G>A); two patients were heterozygous for *c.*3435C>T, and one was a homozygous carrier of the *ABCB1 c.*3435C>T variant [33]. In a previously mentioned study [5], results showed an association of *c*.3435C>T and 1236T-2677T-3435T haplotype with a reduced risk for thromboembolic events with RIVA. Furthermore, 1236C-2677G-3435C and 1236T-2677G-3435C haplotypes are associated with an increased risk for thromboembolic events. 

The ABCG2 multidrug transporter protein is coded by the *ABCG2* gene. EMA and FDA point to the important role of ABCG2 in drug–drug interactions [37,38]. *ABCG2* polymorphisms can decrease the function of ABCG2 transport protein, decrease elimination, and consequently elevate the concentration of substrate drugs. Higher concentrations of drug substrates lead to a greater risk for ADRs. The most significant *ABCG2* polymorphism is *c*.421C>*A*, and the *A* allele is associated with poorer function, i.e., transport of ABCG2 substrate drugs [39,40,41].

A recently published retrospective cohort study found no association of the same eight pharmacogenetic variants investigated with a risk of bleeding from rivaroxaban and apixaban. However, the authors explained the possible reasons for that lack of significant result. One of these reasons is that they did not investigate the polygenic score, i.e., the effect of multiple genetic variants, or the interaction of clinical and pharmacogenetic factors [42].

## 4. Conclusions

In conclusion, we should be aware that pharmacogenetic data have some impact on RIVA pharmacokinetics. NOACs are not free of DDIs, and DDIs have an impact on the risk of bleeding as an adverse event other than age and decreased renal function. We should keep in mind the complexity of NOACs patients’ therapy (an average of 8 drugs in this series) and consider DDIs, but also drug–drug-gene interactions, as drugs can have an inhibitor role and not only be substrates of shared enzymes and transporters of metabolic pathways. For these patientsm comprehensive and systematic management of medications by a trained pharmacist is also crucial.

Our data suggest a possible role of clinical and pharmacogenetic factors and their interactions in predicting bleeding on rivaroxaban treatment; however, they also indicate the need for further comprehensive multidisciplinary research (involving clinicians, pharmacists, and laboratory specialists) to enable safer use of this medicine based on a personalised approach. 

## Figures and Tables

**Table 1:** Demographic, laboratory, and medical data for all subjects.

N	Age	Sex	Dg	*CYP3A4*		*CYP3A5*	*CYP2J2*		*MDR1*/*ABCB1*				*ABCG2*/ *BCRP*	RIVA Adverse Event (Bleeding)	RIVA DD (mg)	eGFR (mL/min /1.73 m^2^)	DDI RR/DDI	DDI Bleeding Risk/RIVA Conc.
				*1B	*22	*3	*7	rs 11572325	c.1236C>T rs 1128503	c.2677G>T rs 2032582	c.3435C>T rs 1045642	rs 4148738	*c*.421C>A					
**1**	68	M	AF	*1/*1	*1/*1	*3/*3	*1/*1	A/A	C/C	G/G	C/C	**G/G**	C/C	GI	20	88	-	-
**2**	80	F	AF	*1/*1	*1/*1	*3/*3	*1/*1	A/A	**T/T**	**T/T**	**T/T**	A/A	C/**A**	GI	15	**38**	C./RIVA-duloxetine D./RIVA-clopidogrel	Bleeding risk may be increased
**3**	64	M	AF	*1/*1	*1/*1	*3/*3	*1/*1	A/A	C/C	G/G	C/C	**G/G**	C/C	GI	20	100	-	-
**4**	75	M	AF	*1/*1	*1/*1	*3/*3	*1/*1	A/A	C/**T**	G/**T**	C/**T**	**G**/A	C/C	GI	20	**43**	D./RIVA-indomethacin	Bleeding risk may be increased
**5**	66	M	AF	*1/*1	*1/*1	*1/*3	*1/*1	A/A	C/**T**	G/**T**	C/**T**	**G**/A	C/C	Haematuria	15	89	B./RIVA-propafenone	P-gp/ABCB1 inhibitors may increase the serum RIVA conc.
**6**	66	F	AF	*1/*1	*1/*1	*3/*3	*1/*1	A/A	C/C	G/G	C/C	**G/G**	C/C	Epistaxis	20	105	-	-
**7**	75	F	AF	*1/*1	*1/*1	*1/*3	*1/*7	A/**T**	C/**T**	G/**T**	C/**T**	G/**A**	C/C	Epistaxis	15	**35**	D./RIVA-ASA D./RIVA-ketoprofen	Bleeding risk may be increased
**8**	72	F	AF	*1/*1	*1/*1	*1/*3	*1/*7	A/**T**	C/C	G/G	C/C	**G/G**	C/**A**	Epistaxis	20	88	-	-
**9**	75	M	AF	*1/*1	*1/*1	*3/*3	*1/*1	A/**T**	C/**T**	G/**T**	C/**T**	**G**/A	C/C	GI	15	**54**	B./RIVA-amiodarone	P-gp/ABCB1 inhibitors may increase the serum RIVA conc.
**10**	76	F	DVT	*1/*1	*1/*1	*3/*3	*1/*1	A/A	C/C	G/G	C/C	**G/G**	C/**A**	GI	15	**34**	-	-
**11**	61	F	DVT	*1/*1	*22/*22	*3/*3	*1/*7	A/**T**	C/**T**	G/**T**	C/**T**	**G**/A	C/C	Gynaecological	10	85	D./RIVA-ASA	Bleeding risk may be increased
**12**	75	F	AF	*1/*1	*1/*1	*3/*3	*1/*1	A/**T**	C/**T**	G/**T**	C/**T**	**G**/A	C/C	GI	15	61	B./RIVA-amiodarone D./RIVA-clopidogrel	P-gp/ABCB1 inhibitors may increase the serum RIVA conc. Bleeding risk may be increased
**13**	78	M	PAD	*1/*1	*1/*1	*3/*3	*1/*7	A/**T**	**T/T**	**T/T**	**T/T**	A/A	C/C	Epistaxis	5	70	D./RIVA-ASA	Bleeding risk may be increased
**14**	74	F	PAD	*1/*1	*1/*1	*3/*3	*1/*1	A/A	C/**T**	G/G	C/*T*	**G/G**	C/C	GI	20	**48**	B./RIVA-amiodarone	P-gp/ABCB1 inhibitors may increase the serum RIVA conc.
**15**	69	F	AF	*1/*1	*1/*1	*3/*3	*1/*1	A/A	C/**T**	G/**T**	C/**T**	**G**/A	C/C	Epistaxis	20	82	-	-
**16**	66	M	AF	*1/*1	*1/*22	*3/*3	*1/*1	A/A	C/C	G/G	C/C	**G**/A	**A/A**	GI	20	63	-	-

AF atrial fibrillation; ASA acetylsalicylic acid; DD daily dose; DDI drug–drug interaction; DVT deep vein thrombosis; eGFR estimated glomerular filtration rate; GI gastrointestinal; PAD peripheral arterial disease; RIVA rivaroxaban; RR risk rating from Lexicomp (B No action needed; C Monitor therapy; D Consider therapy modification). Minor and variant pharmacogenes alleles and decreased eGFR values are in bold.

**Table 2:** Concomitant drugs and their relationship with the rivaroxaban metabolic pathway and possible mechanisms and severity of interactions.

DRUG	CYP3A4	CYP3A5	CYP2J2	MDR1/ABCB1	ABCG2/BCRP	POSSIBLE MECHANISM OF DDI	RIVA CONC.	SEVERITY OF DDI	SOURCE
**Amiodarone**	Substrate, Inhibitor		Inhibitor	Inhibitor		Decreased metabolism	Increased	**MAJOR**	1, 2
**Acetylsalicylic acid**				Substrate, Inducer		Increased anticoagulant activity	-	**MODERATE**	1
**Bisoprolol**	Substrate			Substrate, Inhibitor		--	Increased		
**Clopidogrel**		Substrate		Substrate		Increased anticoagulant activity	-		
**Digoxin**				Substrate, Inhibitor, Inducer		Decreased renal excretion rate	Increased		1, 2
**Duloxetine**	Inhibitor			Inhibitor		Increased anticoagulant effect	-		1
**Eplerenone**	Substrate	Substrate				Increased renal excretion rate (induces diuresis)	Decreased		1, 2
**Fexofenadine**				Substrate		Competition for the P-glycoprotein	Increased		
**Furosemide**	Substrate					Increased renal excretion rate (induces diuresis)	Decreased		1
**Hydrochloro-thiazide**									
**Indapamide**	Substrate								
**Indomethacin**				Substrate, inhibitor		Increased anticoagulant effect	-		
**Ketoprofen**							-		
**Pantoprazole**	Substrate			Substrate, Inhibitor	Substrate, Inhibitor	-	Increased		
**Propafenone**				Inhibitor		-			
**Spironolactone**				Inducer		Increased renal excretion rate (induces diuresis)	Decreased		
**Acetaminophen**	Substrate, Inducer			Substrate, Inducer		Decreased renal excretion rate	Increased	**MINOR**	1
**Allopurinol**					Substrate				
**Alprazolam**	Substrate	Substrate							1, 2
**Atorvastatin**				Substrate, Inhibitor	Substrate	Competition for metabolism			
**Chloroquine**						Decreased renal excretion rate			1
**Diazepam**	Substrate, Inhibitor			Substrate					
**Febuxostat**					Inhibitor	Inhibition of BCRP-mediated efflux			
**Finasteride**	Substrate	Substrate				Competition for metabolism			
**Folic acid**					Substrate	Decreased renal excretion rate			
**Isosorbide mononitrate**									
**Lercanidipine**	Substrate, Inhibitor	Substrate				Competition for metabolism			
**Lorazepam**	Substrate					Decreased renal excretion rate			
**Metformin**									
**Metoprolol**	Substrate								
**Perindopril**									
**Prednisone**	Substrate, Inducer	Inducer		Substrate, Inducer					
**Rosuvastatin**					Substrate	-			1, 2
**Tamsulosin**	Substrate					Decreased renal excretion rate			1
**Tramadol**									

1 DrugBank; 2 FDA DDI Table; DDI drug–drug interaction; RIVA rivaroxaban.

**Table 3:** Subjects’ data with the pharmacogene variant ABCB1 rs4148738GG.

N	Sex	Age	Diagnosis	Therapy (DD)	Adverse Event
1	M	68	AF Anaemia sideropenica AH Hyperlipidemia	RIVA (20 mg) Amlodipine (5 mg) Bisoprolol (5 mg BID) Diazepam (5 mg) Iron(II) fumarate Pantoprazole (40 mg) Perindopril/Indapamide (5/1.25 mg) Rosuvastatin (20 mg)	Gastrointestinal bleeding 6 months following the introduction of RIVA
2	M	64	AF Anaemia sideropenica AH Asthma Diabetes mellitus Extreme obesity Hyperlipidemia Post NSTEMI Stenosis aortae	RIVA (20 mg) Atorvastatin (40 mg) Doxazosin (2 mg) Furosemide (40 mg) Lercanidipine (10 mg BID) Metformin (3 × 1000 mg) Moxonidine (0.6 mg) Nebivolol (2.5 mg) Pantoprazole (40 mg) Spironolactone (50 mg)	Bleeding 1 year (melena) following the introduction of RIVA
3	F	66	AF Sjogren syndrome	RIVA (20 mg) Bisoprolol (2.5 mg) Methyl digoxin (0.1 mg)	Epistaxis 9 months following the introduction of RIVA

AF atrial fibrillation; AH arterial hypertension; DD daily dose; NSTEMI non-ST-elevation myocardial infarction; RIVA rivaroxaban.

**Table 4:** Subjects’ data with several pharmacogene variants (one risk factor).

N	Sex	Age	Diagnosis	Therapy (DD)	Adverse Event	Pharmacogenetic Results/Phenotype
1	F	72	AF AH COPD Hyperlipidemia Hyperuricemia	RIVA (20 mg) Bisoprolol (1.25 mg) Eplerenone (25 mg) Furosemide (40 mg) Perindopril butylamine/ Indapamide (4/12.5 mg)	Epistaxis 4 months following the introduction of RIVA	CYP2J2 *1/*7 decreased enzyme activity CYP3A5 *1/*3 expresser ABCB1 (MDR1) 1236-2677-3435-rs4148738 CC-GG-CC-**GG** risk allele ABCG2 421C**A** decreased function
2	F	69	AF AH Hypothyroidism PAD	RIVA (20 mg) Atorvastatin (20 mg) Bisoprolol 1.25 mg) Diazepam (2 mg) Levothyroxine (100 mg) Perindopril/Indapamide/Amlodipine (10/2.5/5 mg)	Epistaxis 5 months following the introduction of RIVA	ABCB1 (MDR1) 1236-2677-3435-rs4148738 C**T**-G**T**-C**T**-**G**A intermediate function
3	M	66	AF Anaemia sideropenica CAD Colon polyps Post STEMI	RIVA (20 mg) Atorvastatin (40 mg) Perindopril/Amlodipine (10/10 mg) Trimetazidine (2 × 35 mg) Nebivolol (5 mg) Moxonidine (0.4 mg) Furosemide (40 mg) Pantoprazole (20 mg)	Gastrointestinal bleeding and anaemia 4 months following the introduction of RIVA	CYP3A4 *1/*22 decreased enzyme activity ABCB1 (MDR1) 1236-2677-3435-rs4148738 CC-GG-CC-**G**A risk allele ABCG2 421**AA** poor function

AF atrial fibrillation; AH arterial hypertension; CAD coronary artery disease; COPD Chronic obstructive pulmonary disease; DD daily dose; PAD peripheral artery disease RIVA rivaroxaban; STEMI ST-elevation myocardial infarction.

**Table 5:** Data of subjects with known possible DDI plus pharmacogene variants (two risk factors).

N	Sex	Age	Diagnosis	Therapy (DD)	Bleeding	Pharmacogenetic Results/ Phenotype	DDI (Lexicomp)
1	M	66	AF Anaemia sideropenica AH Benign prostatic hyperplasia Diabetes mellitus type 2 Guillain Barre syndrome Hyperlipidemia	**RIVA** (15 mg) Calcitriol (0.5 mcg) Diazepam (5 mg) Finasteride (5 mg) Folate (5 mg) Furosemide (40 mg) Metoprolol (25 mg) Methylprednisolone (4 mg) Pantoprazole (20 mg) **Propafenone** (150 mg, 1+0+2) Tamsulosin 0.4 mg Zolpidem (5 mg)	Macroscopic haematuria relapse	CYP3A5 *1/*3 expresser ABCB1 (MDR1) 1236-2677-3435-rs4148738 C**T**-G**T**-C**T**-**G**A intermediate function	RIVA—propafenone
2	F	61	AH DVT Hyperlipidemia Thrombophilia (heterozygosity for the Leiden variant)	**RIVA** (10 mg) **ASA** (100 mg) Bisoprolol (2.5 mg) Ezetimibe (10 mg) Moxonidine (0.4 mg) Pantoprazole (40 mg) Perindopril/Indapamide (4/1.25 mg) Rosuvastatin (40 mg)	Extreme gynaecological bleeding 1 month following the introduction of RIVA	CYP2J2 *1/*7 decreased enzyme activity CYP3A4 *22/*22 poor enzyme activity ABCB1 (MDR1) 1236-2677-3435-rs4148738 C**T**-G**T**-C**T**-**G**A intermediate function	RIVA—ASA
3	F	75	AF AH Liver lesion Post NSTEMI	**RIVA** (15 mg) **Amiodarone** (200 mg) Bisoprolol (1.25 mg) **Clopidogrel** (75 mg) Empagliflozin (10 mg) Eplerenone (25 mg) Furosemide (40 mg) Metformin (500 mg bid) Pantoprazole (40 mg) Perindopril/Amlodipine (5/5 mg) Rosuvastatin (40 mg)	Melena	ABCB1 (MDR1) 1236-2677-3435-rs4148738 C**T**-G**T**-C**T**-**G**A intermediate function	RIVA—amiodarone RIVA—clopidogrel
4	M	78	AH Anaemia sideropenica Aortic coronary stent Hyperlipidemia PAD	**RIVA** (5 mg) Alirocumab (150 mg) two times monthly Amlodipine/Valsartan (10/160/12.5 mg BID) **ASA** 100 mg Atorvastatin (80 mg) Ezetimibe (10 mg)	Epistaxis 6 months following the introduction of RIVA	CYP2J2 *1/*7 decreased enzyme activity ABCB1 (MDR1) 1236-2677-3435-rs4148738 **TT**-**TT**-**TT**-AA poor function	RIVA—ASA

AF atrial fibrillation; AH arterial hypertension; ASA acetylsalicylic acid; DD daily dose; DDI drug–drug interaction; DVT deep vein thrombosis; NSTEMI non-ST-elevation myocardial infarction; PAD peripheral arterial disease; RIVA rivaroxaban.

**Table 6:** Data of subjects with known decreased kidney function (eGFR < 60 mL/min/1.73 m^2^) plus pharmacogene variants (two risk factors).

N	Sex	Age	Diagnosis	Therapy (DD)	Adverse Event	Kidney Function	Pharmacogenetic Results/Phenotype
1	F	76	AH Arterial depression GERD Heart failure Post DVT Rheumatoid arthritis	RIVA (15 mg) Alprazolam Febuxostat Lorazepam Mirtazapine Hydroxychloroquine Pantoprazole (40 mg) Prednisone Ramipril	Anaemia, melena 4 months following the introduction of RIVA	**eGFR = 34** mL/min/1.73 m^2^ (decreased kidney function)	ABCB1 (MDR1) 1236-2677-3435-rs4148738 CC-GG-CC-**GG** risk allele ABCG2 421C**A** decreased function

AH arterial hypertension; DD daily dose; DVT deep vein thrombosis; eGFR estimated glomerular filtration rate; GERD gastroesophageal reflux disease; RIVA rivaroxaban.

**Table 7:** Data of subjects with known decreased kidney function (eGFR < 60 mL/min/1.73 m^2^), plus possible DDI plus pharmacogene variants (three risk factors).

N	Sex	Age	Diagnosis	Therapy (DD)	Adverse Event	Pharmacogenetic Results/ Phenotype	Kidney Function	DDI (Lexicomp)	
1	F	80	AF AH Glaucoma Hyperlipidaemia Ischemic cardiomyopathy Post STEMI	**RIVA** (15 mg) Atorvastatin (80 mg) Bisoprolol (2.5 mg) **Clopidogrel** (75 mg) **Duloxetine** (30 mg) Eplerenone (25 mg) Furosemide (40 mg) Pantoprazole (40 mg) Ramipril (2.5 mg) Tramadol/ Paracetamol (50 mg)	GI bleeding (melena) 3 months following the introduction of RIVA	ABCB1 (MDR1) 1236-2677-3435-rs4148738 **TT**-**TT**-**TT**-AA poor function ABCG2 421C**A** decreased function	**eGFR = 38** mL/min/1.73 m^2^ (decreased kidney function)	RIVA-clopidogrel RIVA-duloxetine	
2	M	75	AF Anaemia sideropenica AH Gout Hyperuricemia	**RIVA** (15 mg) Allopurinol (100 mg) Bisoprolol (1.25 mg) **Indomethacin** (50 mg bid) Pantoprazole (40 mg) Perindopril/ Indapamide (4/1.25 mg)	GI bleeding and anaemia 1 year following the introduction of RIVA	ABCB1 (MDR1) 1236-2677-3435-rs4148738 C**T**-G**T**-C**T**-**G**A intermediate function	**eGFR = 43 mL**/min/1.73 m^2^ (decreased kidney function)	RIVA- indomethacin	
3	F	75	AF Aortic stenosis AH CAD Chronic kidney disease Diabetes mellitus type II Glaucoma Thyroid struma	**RIVA** (15 mg) Allopurinol (100 mg) Amlodipine/Valsartan/Hydrochlorothiazide (5/160/12.5 mg) **ASA** (100 mg) Atorvastatin (40 mg) Bisoprolol (1.25 mg) Diazepam (5 mg) Dulaglutide (1.5 mg once weekly) Fexofenadine (120 mg) Furosemide 40 mg Insulin basal 18 i.u.s.c **Ketoprofen** 150 mg Levothyroxine 25 mcg Pantoprazole 40 mg Repaglinide 2 mg Trimetazidine (35 mg BID)	Epistaxis 3 months following the introduction of RIVA	CYP2J2 *1/*7 decreased enzyme activity CYP3A5 *1/*3 expresser ABCB1 (MDR1) 1236-2677-3435-rs4148738 C**T**-G**T**-C**T**-**G**A intermediate function	**eGFR = 35 mL**/min/1.73 m^2^ (decreased kidney function)	RIVA-ASA RIVA-ketoprofen	
4	M	75	AF Anaemia sideropenica AH Nicotinism	**RIVA** (15 mg) **Amiodarone** (200 and 100 mg alternately) Pantoprazole (40 mg) Perindopril (4 mg)	GI bleeding (melena)	ABCB1 (MDR1) 1236-2677-3435-rs4148738 C**T**-G**T**-C**T**-**G**A intermediate function	**eGFR = 54** mL/min/1.73 m^2^ (decreased kidney function)	RIVA-amiodarone	
5	F	74	AF Anaemia sideropenica AH Artificial cardiac pacemaker CAD Diabetes mellitus type II Hyperlipidaemia PAD	**RIVA** (15 mg) Atorvastatin (20 mg) **Amiodarone** (100 mg) Trandolapril (4 mg) Metformin (2 × 850 mg) Isosorbide mononitrate (40 mg) Insulin	GI bleeding and anaemia	ABCB1 (MDR1) 1236-2677-3435-rs4148738 C**T**-GG-C**T**-**GG** **risk alleles**	**eGFR = 48 mL**/min/1.73 m^2^ (decreased kidney function)	RIVA-amiodarone	

AF atrial fibrillation; AH arterial hypertension; ASA acetylsalicylic acid; CAD coronary artery disease; DD daily dose; DDI drug–drug interaction; GI gastrointestinal; PAD peripheral arterial disease; RIVA rivaroxaban; STEMI ST-elevation myocardial infarct.

## Data Availability

The data that support the findings of this study are available from the corresponding author upon reasonable request. The study is registered on ClinicalTrials.gov (NCT05307718).

## Appendix A

### Study Design and Methodology

The study “Pharmacogenomics in Prediction of Cardiovascular Drugs Adverse Reaction—PGx CardioDrug” is registered on ClinicalTrials.gov; the identifier is NCT05307718. The study has been ongoing since December 2020, and till now, 762 patients have been recruited.
